# Genome Sequence of *Kocuria polaris* Strain CD08_4, an Isolate from the Duodenal Mucosa of a Celiac Disease Patient

**DOI:** 10.1128/genomeA.01158-17

**Published:** 2017-10-26

**Authors:** Atul Munish Chander, Munesh Kumari, Rakesh Kochhar, Devinder Kumar Dhawan, Sanjay Kumar Bhadada, Shanmugam Mayilraj

**Affiliations:** a Department of Biophysics, Panjab University, Chandigarh, India; b Microbial Type Culture Collection and Gene bank (MTCC), CSIR, Institute of Microbial Technology, Chandigarh, India; c Department of Gastroenterology, Postgraduate Institute of Medical Education and Research (PGIMER), Chandigarh, India; d Department of Endocrinology, Postgraduate Institute of Medical Education and Research (PGIMER), Chandigarh, India

## Abstract

We report here the 3.8-Mb genome sequence of *Kocuria polaris* strain CD08_4, an isolate from the duodenal mucosa of a celiac disease patient. The genome consists of specific virulence determinant genes, antibiotic resistance genes, genes for coping with oxidative stress, and genes responsible for iron acquisition and metabolism, suggestive of its pathogenic attributes.

## GENOME ANNOUNCEMENT

At least 100 trillion microbes inhabit the human body (1) and play a significant role in human health and disease. Celiac disease (CD) is an autoimmune disease in which intestinal microbial dysbiosis is associated with disease presentations (2). This is the first genome sequence report of *Kocuria polaris* from a clinical source, which may help understand the molecular basis of its pathogenic behavior for the future. Previously, we reported *Kocuria palustris* CD07_3 from a CD patient, and *K. polaris* CD08_4 is another species of the genus (3) that was isolated from the duodenal mucosa of a CD patient who was tTG IgA antibody (Ab) positive (128 U/ml) and presented with short stature and anemia. The patient was recruited at the Postgraduate Institute of Medical Education and Research (PGIMER), Chandigarh, India. The study was approved by the ethics committee of PGIMER, and informed written consent was obtained from the participant.

Genomic DNA was extracted from a 48-h-old culture using the ZR fungal/bacterial DNA miniprep kit. Strain CD08_4 was identified as *Kocuria polaris* by 16S rRNA gene sequencing. The genome of *K. polaris* strain CD08_4 was sequenced using Illumina HiSeq 1000 technology. A total of 17,179,087 reads were generated, having 1,630,265,732 bp, and were *de novo* assembled into 42 contigs using CLC Genomics Workbench version 7.5.1 (CLC bio, Aarhus, Denmark). The assembled genome of strain CD08_4 had a total length of 3,833,718 bp, mean coverage of 100×, an *N*_50_ value of 221,530 bp, and a mean G+C content of 72.6%.

The Rapid Annotations using Subsystems Technology (RAST) server was used for functional annotation of the genome (4–6), and the genome was predicted to contain 3,635 coding sequences in 346 subsystems, along with 52 RNAs. The genomic annotations in the genome database of NCBI revealed that strain CD08_4 contains 49 tRNAs, 3 rRNAs, 1 noncoding RNA (ncRNA), and 95 pseudogenes. We highlighted some important genes of clinical relevance, so infections with this microbe may a require clinician’s attention. Forty genes were present in the categories of virulence, disease, and defense. Strain CD08_4 contains genes coding for a multidrug resistance transporter of the Bcr/CflA family and mutated genes coding for enzyme DNA gyrase subunit B, DNA gyrase subunit A, topoisomerase IV subunit B, and topoisomerase IV subunit A that can cause resistance against fluoroquinolones. The strain also had genes coding for β-lactamases, class C β-lactamases, and other penicillin-binding proteins that are the causal genes for resistance against some antibiotics in the β-lactam class. As highlighted previously (7), the iron acquisition genes and oxidative stress genes can play a putative role in CD pathogenesis; strain CD08_4 was also reported to contain 7 genes responsible for iron acquisition and metabolism and 24 genes to cope with oxidative stress. Among 24 genes, *K. polaris* CD08_4 had 3 genes coding for enzyme peroxidase, catalase, and superoxide dismutase, which may provide protection against reactive oxygen species generated during host-microbe interactions, thereby leading to self-protection.

### Accession number(s).

This whole-genome shotgun project has been deposited at DDBJ/EMBL/GenBank under the accession number LQBK00000000. The version described in this paper is the first version, LQBK01000000.
